# Prognostic utility of serum free light chain ratios and heavy-light chain ratios in multiple myeloma in three PETHEMA/GEM phase III clinical trials

**DOI:** 10.1371/journal.pone.0203392

**Published:** 2018-09-07

**Authors:** Lucia Lopez-Anglada, Cecilia Cueto-Felgueroso, Laura Rosiñol, Albert Oriol, Ana Isabel Teruel, Ana Lopez de la Guia, Enrique Bengoechea, Luis Palomera, Felipe de Arriba, Jose Mariano Hernandez, Miquel Granell, Francisco Javier Peñalver, Ramon Garcia-Sanz, Juan Besalduch, Yolanda Gonzalez, Rafael Benigno Martinez, Miguel Teodoro Hernandez, Norma C. Gutierrez, Paloma Puerta, Antonio Valeri, Bruno Paiva, Joan Blade, Maria-Victoria Mateos, Jesus San Miguel, Juan Jose Lahuerta, Joaquin Martinez-Lopez, on behalf of the GEM (Grupo Español de MM)/PETHEMA (Programa para el Estudio de la Terapéutica en Hemopatías Malignas) Cooperative Study Group

**Affiliations:** 1 Hematology Department, Hospital Universitario 12 de Octubre, CIBERONC, Madrid, Spain; 2 Clinical Biochemistry Department, Hospital Universitario 12 de Octubre, Madrid, Spain; 3 Hematology Department, Hospital Clinic, Institut d'Investigacions Biomédiques August Pi I Sunyer (IDIBAPS), Barcelona, Spain; 4 Hospital Germans i Trials, Barcelona, Spain; 5 Hematology Department, Hospital Clínico de Valencia, Valencia, Spain; 6 Hematology Department, Hospital La Paz, Madrid, Spain; 7 Hematology Department, Hospital de Donostia, San Sebastian, Spain; 8 Hospital Clínico Universitario Lozano Blesa, Zaragoza, Spain; 9 Hospital Morales Meseguer, Murcia, Spain; 10 Hematology Department, Hospital General de Segovia, Segovia, Spain; 11 Haematology, Hospital Univarsitari de la Santa Creu i Sant Pau, Barcelona,Spain; 12 Haematology Department, Hospital Universitario Fundacion Alcorcon, Madrid, Spain; 13 Hematology Department, Hospital Universitario de Salamanca-IBSAL, IBMCC-CSIC, Salamanca, Spain; 14 Haematology Department, Hospital Univarsitari Son Espases, Mallorca, Spain; 15 Haematology Department, Institutd’Oncologia Dr Josep Trueta de Girona, Girona, Spain; 16 Haematology Department, Hospital Universitario San Carlos, Madrid, Spain; 17 Haematology Department, Hospital Universitario de Canarias, Tenerife, Spain; 18 Haematology Department, Clínica Universitaria de Navarra/ CIMA,IDISNA, CIBERONC,Pamplona, SPAIN; Cleveland Clinic, UNITED STATES

## Abstract

We investigated the prognostic impact and clinical utility of serum free light chains (sFLC) and serum heavy-light chains (sHLC) in patients with multiple myeloma treated according to the GEM2005MENOS65, GEM2005MAS65, and GEM2010MAS65 PETHEMA/GEM phase III clinical trials. Serum samples collected at diagnosis were retrospectively analyzed for sFLC (*n* = 623) and sHLC (*n* = 183). After induction or autologous transplantation, 309 and 89 samples respectively were available for sFLC and sHLC assays. At diagnosis, a highly abnormal (HA) sFLC ratio (sFLCr) (<0.03 or >32) was not associated with higher risk of progression. After therapy, persistence of involved-sFLC levels >100 mg/L implied worse survival (overall survival [OS], *P* = 0.03; progression-free survival [PFS], *P* = 0.007). Among patients that achieved a complete response, sFLCr normalization did not necessarily indicate a higher quality response. We conducted sHLC investigations for IgG and IgA MM. Absolute sHLC values were correlated with monoclonal protein levels measured with serum protein electrophoresis. At diagnosis, HA-sHLCrs (<0.29 or >73) showed a higher risk of progression (*P* = 0.006). Additionally, involved-sHLC levels >5 g/L after treatment were associated with shorter survival (OS, *P* = 0.001; PFS, *P* = 0.018). The HA-sHLCr could have prognostic value at diagnosis; absolute values of involved-sFLC >100 mg/L and involved-sHLC >5 g/L could have prognostic value after treatment.

## Introduction

The immunoassay for quantifying free immunoglobin light chains in serum (sFLC) has been a valuable tool for diagnosing and monitoring light-chain multiple myeloma (MM) [1], amyloid light-chain amyloidosis, [2], and monoclonal gammopathies of undetermined significance (MGUS) [3,4]. Moreover, the recently developed assays for quantifying specific immunoglobin heavy-light chain complexes in serum (sHLC) have enabled accurate measurements of the different types of light chains (termed κ and λ) coupled with a specific heavy chain (i.e., IgA, IgG, or IgM) in intact immunoglobulins. Thus, the ratios of heavy-light chain-κ to heavy-light chain-λ can be determined (e.g., the IgG-κ:IgG-λ ratio). The latter assay has made it possible to measure isotype-specific suppression of the sHLC pair that is not involved with the disease, such as the suppression of IgA-λ in a patient with IgA-κ type MM [5,6].

The sFLC assays complement the classic assays of serum protein electrophoresis (sPE) and immunofixation, which increases the diagnostic sensitivity of serum protein studies in MM [4]. In addition to its diagnostic utility [3], an abnormal sFLC ratio (sFLCr) is a risk factor for progression from MGUS, smoldering myeloma, or solitary plasmacytoma to symptomatic MM. However, the prognostic role of sFLC in symptomatic MM remains unclear [1,4,7,8,9,10,11]. Some studies have reported a relationship between baseline levels of the involved-sFLC level or the sFLCr and the disease burden; however, other studies have not found any relationship [1,4,7,9,10,11].

The normalization of sFLCr has been incorporated into the consensus definition of a stringent complete response (CR) [12,13,14]. However, a previous analysis conducted by our group failed to demonstrate a prognostic role for normal sFLCrs in the CR [15]. The short half-lives of κ and λ FLCs may make them a reliable indicator of the response to treatment, but their clinical utility in this context remains under investigation. Moreover, sFLCs are likely to be more useful than conventional methods for diagnosing light-chain MM [16].

A recent report [17] showed that quantification with the IgA sHLC assay was more accurate than either an M-protein quantification with sPE or a total IgA quantification with nephelometry. Thus, the sHLC assay could provide a solution to some issues associated with co-migration in electrophoresis, particularly in cases of oligosecretory MM. However, only a few published reports [18,19] and several small case series have addressed the use of sHLC analyses for evaluating patients with MM.

In the present study, we extended our previous series [15] to investigate the utility and prognostic impact of the sFLCr and the sHLCr. We measured the levels of involved-sFLC and involved-sHLC at diagnosis and after therapy in patients with MM treated in the PETHEMA/GEM phase III clinical trials.

## Patients and methods

### Patients and treatment

All patients were included and treated according to the protocols implemented in three PETHEMA/GEM (Programa para el Estudio de la Terapéutica en Hemopatías Malignas/Grupo Español de Mieloma) phase III clinical trials [20,21,22]. In brief, in the GEM2005MENOS65 trial (NCT00461747), 390 patients (130 per arm) were randomly assigned to receive one of the following induction therapies: (arm 1: VCMP/VBAD/B) vincristine, BCNU, melphalan, cyclophosphamide, prednisone (VCMP)/ vincristine, BCNU, doxorubicin (Adriamycin®), and dexamethasone (VBAD), followed by two courses of bortezomib (B); (arm 2: TD) thalidomide and dexamethasone or (arm 3: VTD) bortezomib, thalidomide, and dexamethasone. All patients were scheduled to undergo autologous stem cell transplantation with high-dose melphalan, followed by maintenance therapy with interferon alpha-2b, thalidomide, or thalidomide plus one cycle of bortezomib, every 3 months for up to 3 years. In the GEM2005MAS65 trial (NCT00443235), 260 patients were randomly assigned to receive six cycles of one of the following induction therapies: (arm 1: VMP) bortezomib, melphalan, and prednisone or (arm 2: VTP) bortezomib, thalidomide, and prednisone. This treatment was followed by maintenance with bortezomib and thalidomide or bortezomib and prednisone for up to 3 years. In the GEM2010MAS65 trial (NCT01237249), 260 patients >65 years old were randomly assigned to receive 18 cycles of the following induction therapies: (arm 1) nine cycles of VMP, followed by nine cycles of lenalidomide and dexamethasone or (arm 2) alternating cycles of VMP and lenalidomide+dexamethasone [22]. All patient serum samples were collected after obtaining informed consent, and the study was conducted in accordance with the local ethics committee (Hospital Universitario 12 de Octubre ethics committee) and the Helsinki Declaration.

### Protein analysis

The sPE assays were conducted with agarose gel electrophoresis (REP; Helena Laboratories, Beaumont, TX, USA) and/or capillary electrophoresis (V8; Helena Biosciences Europe, Gateshead, UK). Serum immunofixation assays were performed to evaluate γ, α, μ, κ, and λ immunoglobulin chains (Hydrasys and Hydragel; Sebia, Lisses, France, and/or SAS-3 and SAS-4, Helena Biosciences Europe).

### sFLC and sHLC analysis

We performed the sFLC assay (FREELITE®; The Binding Site, Birmingham, UK) on an automated nephelometer (BNII; Dade Behring, Marburg, Germany) or a turbidimeter (SPA-PLUS; The Binding Site, Birmingham, UK), with similar results. We quantified IgG-κ, IgG-λ, IgA-κ, and IgA-λ with Hevylite™ reagents provided by the manufacturer in a turbidimeter (SPA-PLUS; The Binding Site). For the sHLC assay, we derived reference ranges for the IgG and IgA sHLC proteins from 30 healthy serum donors that were 50–65 years old and equally balanced for sex; this analysis yielded similar ranges as previously described by other authors (Table 1) [5,6,18].

**Table 1:** Reference ranges for serum IgG and IgA heavy-light chain concentrations, based on samples from healthy individuals (controls) and previously published data.

**Source for IgG controls**	**IgG-K (g/L)**	**IgG-λ (g/L)**	**IgG-K/IgG-λ (95% RI)**
Controls (*n* = 30)	5.45–6.5	2.65–3.25	1.89–2.33
95% RI (min–max)*	(3.16–8.18)*	(1.75–5.28)*	(1.02–3.22)*
IgG HLC product inserts: SPA_PLUS_	3.84–12.07	1.91–6.74	1.12–3.21
IgG HLC product inserts: BNII	4.03–9.78	1.97–5.71	0.98–2.75
Katzmann et al. [5,6]	4.34–10.80	1.77–5.31	1.30–3. 70 [5]/1.17–3.61 [6]
**Source for IgA controls**	**IgA-K (g/L)**	**IgA-λ (g/L)**	**IgA-K/IgA-λ (95% RI)**
Controls (*n* = 30)	1.16–1.50	0.76–0.94	1.43–1.70
RI 95% (min–max)*	(0.61–2.43)*	(0.42–1.42)*	(0.94–2.58)*
IgA HLC product inserts: SPA_PLUS_	0.57–2.08	0.44–2.04	0.78–1.94
IgA HLC product inserts: BNII	0.48–2.82	0.36–1.98	0.80–2.04
Katzmann et al.[5,6]	0.53–2.62	0.38–1.81	0.70–2.20 [5,6]

* (minimum–maximum)

RI, reference interval; HLC, heavy-light chain; SPA_plus_: turbidimeter purchased from The Binding Site (Birmingham, UK); BNII: nephelometer, purchased from Dade Behring (Marburg, Germany)

### Statistical analysis

Survival was analyzed with the Kaplan–Meier method, and statistical significance was tested with the two-sided log-rank test. Progression-free survival (PFS) was measured from the first day of random assignment to the day that disease progression was detected or the day of death from any cause. Overall survival (OS) was measured from the day of first random assignment to the day of death from any cause. A multivariate Cox proportional hazards model (stepwise regression) was developed to explore independent effects on survival. Variables were retained in the model when levels of significance were *P*<0.05. All statistical analyses were performed with SPSS software, version 20.0 (SPSS, Chicago, IL, USA).

## Results

**Fig 1:**
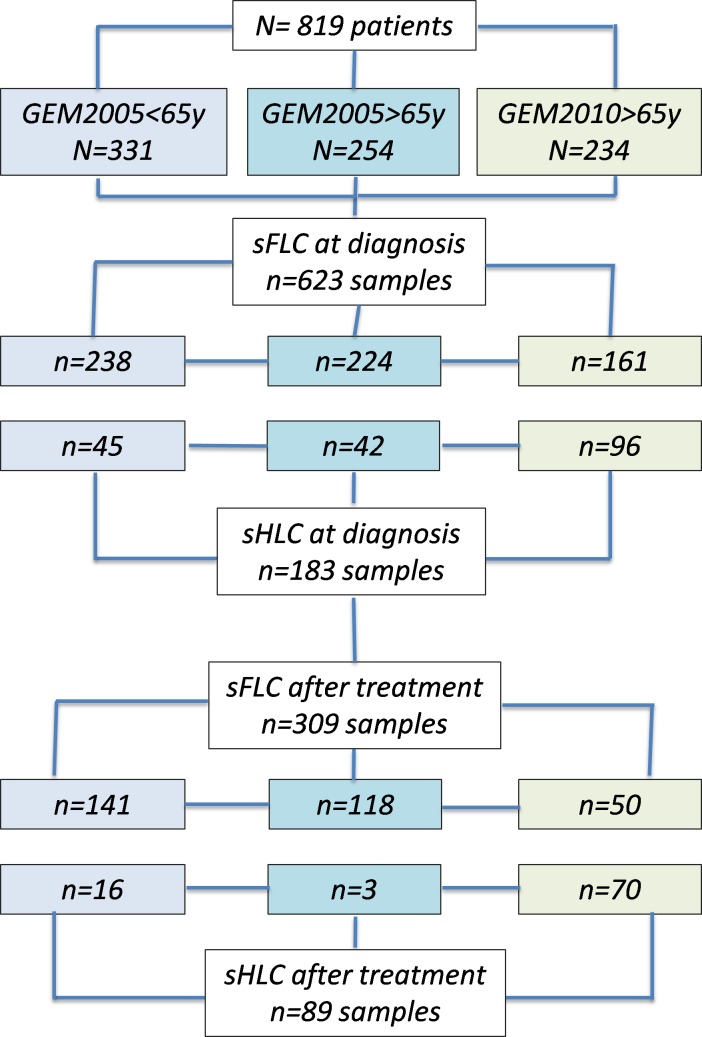
Flow chart: Patient inclusion and analyzed samples.

Of 819 included patients, serum samples for sFLC and sHLC analyses were available at the time of diagnosis in 623 and 183 cases, respectively. After induction (GEM2005MAS65 and GEM2010MAS65) or after autologous transplantation (GEM2005MENOS65), samples were available in 309 cases for sFLC and 89 for sHLC (flow chart, Fig 1). All patients included in the sHLC analysis had either IgG or IgA MM. For the entire series, the median follow-up time was 75 months. S1 Table shows the patient baseline demographics and disease characteristics.

### Prognostic impact of sFLC

**Fig 2:**
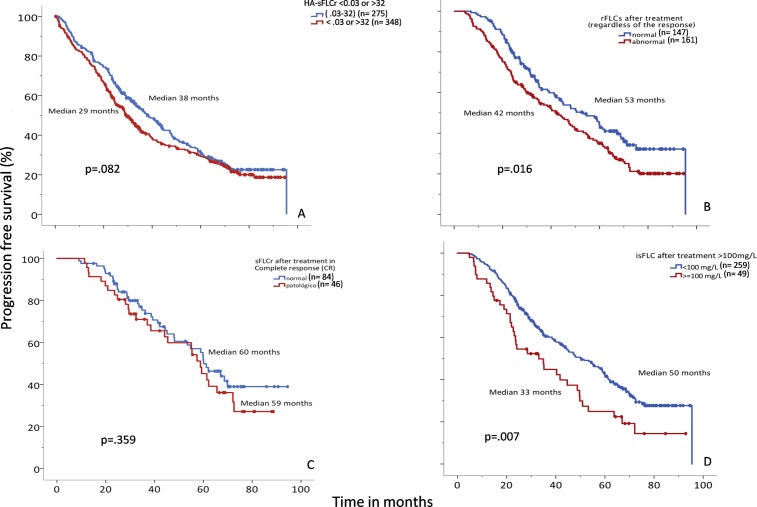
Progression-free survival (PFS) relationships to serum free light chain (sFLC) evaluations. (A) PFS among patients with “highly abnormal” sFLC ratios (<0.03 or >32, red lines, median PFS: 38 mo) compared to those with normal sFLC ratios (0.03–32, blue lines, median PFS: 29 mo). (B) PFS among patients with normal (blue, median PFS: 53 mo) or abnormal (red, median PFS: 42 mo) sFLC ratios after treatment, regardless of the response achieved. (C) PFS among patients that achieved complete response (CR) with normal (blue, median PFS: 60 mo) or abnormal (red, median PFS: 59 mo) sFLC ratios. (D) PFS among patients with absolute involved-sFLC levels <100 mg/L (blue, median PFS: 50 mo) or ≥100 mg/L (red, median PFS: 33 mo).

*sFLCr at diagnosis*: At diagnosis, 92% of the sFLCr values were abnormal. However, when these abnormal values were used to define threshold values, even the highest ratio had no prognostic influence (PFS, *P* = 0.082, Fig 2A; OS, *P* = 0.404, Figure A in S1 Fig). Similarly, absolute involved-sFLC values ≥100 mg/L (or any arbitrary cut-off) measured at diagnosis were not associated with the differences in outcome observed in groups defined by the cutoff.

*sFLCr in post-treatment response assessments*: The prognostic impact of sFLCr normalization is shown in Fig 2B. We observed better survival among patients with normal sFLCr compared to those with abnormal sFLCr values (median PFS, 53 vs. 42 months, *P* = 0.016; median OS, not reached (NR) vs. 80 months, *P* = 0.025, Figure B in S1 Fig). The relationships between normal/abnormal sFLCr and the degree of response are shown in Table 2. As expected, we found that normalization of the sFLCr was associated with a higher degree of response. However, when we analyzed the impact of sFLCr normalization on survival among the 130 patients that achieved CR after induction, we found no significant survival differences (OS, *P* = 0.137; PFS, *P* = 0.359) between patients with normal (0.26–1.65) vs. abnormal sFLCr (Fig 2C; Figure C in S1 Fig).

**Table 2:** Relationship between accepted response categories and sFLCr normalization.

	*No*. *of patients*	sFLCr status*	
*Category of response*		*Normal (%)*	*Abnormal (%)*
***CR***	*130*	*84 (65)*	*46 (35)*
***VGPR***	*24*	*13 (54)*	*11 (46)*
***PR***	*125*	*48 (38)*	*77 (62)*
***SD***	*22*	*2 (9)*	*20 (91)*
***PD***	*8*	*1 (12)*	*7 (88)*
***Total***	*309*	*148*	*161*

*sFLCr normalization was related to a better response (Χ^2^, P<0.0001); however, an analysis of the sFLCr normalization status in each response category showed that a normalized sFLCr did not affect survival.

CR: complete response; VGPR: very good partial response; PR: partial response; SD: stable disease; PD: progressive disease

Next, we explored the prognostic influence of a persistently abnormal value of involved-sFLC after treatment. We found that, when the absolute value of involved-FLCs was ≥100mg/L, it could identify patients at an increased risk of progression; a receiver operating curve (ROC) analysis showed an area under the curve (AUC) of 0.82, a sensitivity of 60%, and a specificity of 89% (P <0.0001)].When we applied these criteria (≥100 or <100 mg/L involved-sFLC) to our data, we found that, after treatment, an absolute value of involved-sFLC ≥100 mg/L was associated with worse survival (median OS, NR vs. 58 months, *P* = 0.03; Figure D in S1 Fig; and median PFS, 50 vs. 33 months, *P =* 0.007; Fig 2D). When we analyzed only patients with a partial response, we found that an absolute value of involved-sFLC ≥100 mg/L after treatment was also associated with a higher risk of progression (median OS, NR vs. 79 months, *P* = 0.158; median PFS, 43 vs. 24 months, *P* = 0.015).

### Prognostic impact of sHLC

**Fig 3:**
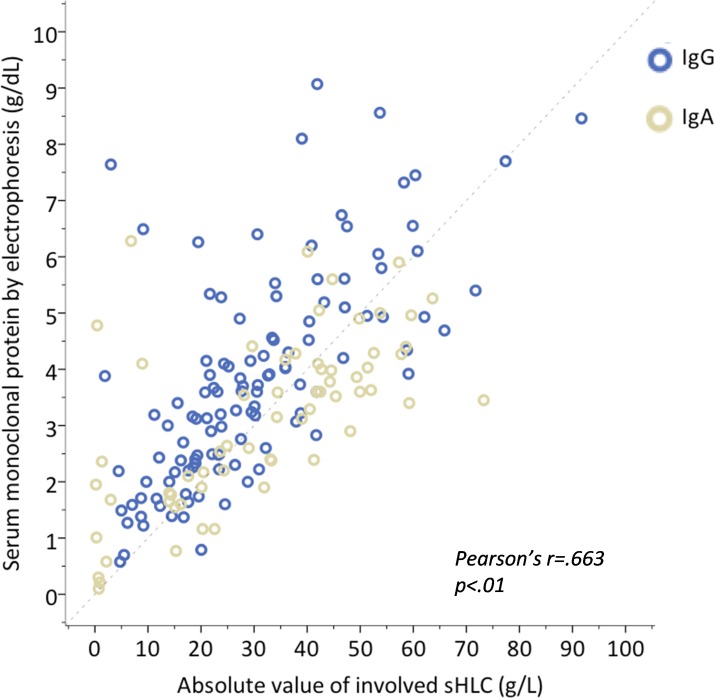
Absolute values of monoclonal proteins (MPs) measured with serum protein electrophoresis (sPE) vs. absolute values of the involved heavy-light chains (isHLC). Correlation analysis shows agreement between the absolute values of IgA (blue, r = 0.65 and IgG (grey, r = 0.71) MPs and isHLC measurements.

*sHLCr at diagnosis*: At diagnosis, the sHLCr was abnormal in 98% of patients. The level of IgG or IgA monoclonal protein (MP), determined with a sHLC analysis, showed a high linear correlation with the absolute values of MP assessed with sPE (*P*<0.001; Pearson's *r* = 0.663) (Fig 3). This correlation was also confirmed after treatment (*P*<0.01; Pearson’s r = 0.692).

**Fig 4:**
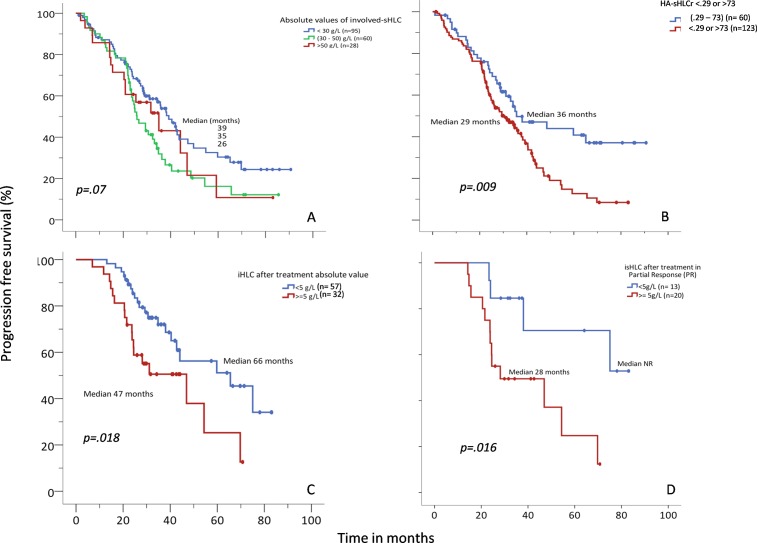
Progression-free survival (PFS) relationships to the absolute value of involved serum heavy-light chain (isHLC) and the ratio of highly abnormal sHLC (HA-sHLCr). (A) PFS among patients with absolute values of isHLC <30 g/L (blue, median PFS: 39 mo), 30–50 g/L (green, median PFS: 26 mo), or >50 g/L (red, median PFS: 35 mo). (B) PFS among patients with HA-sHLCr (<0.29 or >73, red; median PFS: 29 mo) vs. those with normal sHLCr (0.29–73, blue; median PFS: 36 mo). (C) PFS among patients with after-treatment absolute values of isHLC <5 g/L (blue, median PFS: 66 mo) vs. *≥*5 g/L (red, median PFS: 47 mo), regardless of the response achieved. (D) PFS among patients that achieved partial response (PR) after treatment with absolute values of isHLC <5 g/L (blue, median PFS: Non reached (NR) vs. *≥*5 g/L red, median PFS: 28 mo).

We observed that absolute values of involved-sHLC relate to the tumor burden (Fig 4A), and we noted that HA-sHLCr values (<0.29 or >73) at the time of diagnosis were associated with a shorter PFS, (*P* = 0.009; Fig 4B). This finding was confirmed by multivariate analysis, in which an increased risk of progression was associated with the following factors: the HA-sHLCr (odds ratio [OR] 1.78, 95% confidence interval [CI]: 1.14–2.78; *P* = 0.01), age: (OR 1.04, 95% CI: 1.01–1.06; *P* = 0.003); LDH (OR 0.4, 95%CI: 0.26–0.94; *P* = 0.03), and a high- vs. low-risk FISH result (OR 1.75, 95%CI: 1.11–2.74; *P* = 0.02; S2 Table). Although not statistically significant, we noted a trend towards an association between HA involved-sHLC values and a shorter PFS (P = 0.07).

Finally, the suppression of the uninvolved-sHLC pair was not associated with prognosis in our series (OS, *P* = 0.89; PFS, *P* = 0.57).

*sHLCr in post-treatment response assessments*: After treatment, we found no statistically significant association between sHLCr normalization and survival (OS, *P* = 0.841; PFS, *P* = 0.402).

Next, we explored the prognostic value of involved-sHLC that were persistently abnormal after treatment. A ROC curve analysis (AUC 0.859; sensitivity, 100%; specificity, 70%; *P* = 0.002) suggested that involved-sHLC values <5 g/L would be the best cut-off for identifying patients that responded to treatment. Absolute involved-sHLC values ≥5 g/L were associated with shorter survival (OS, NR vs. 57 months, *P* = 0.001; PFS, 66 vs. 47 months, *P* = 0.018; Fig 4C). Moreover, in patients that experienced a partial response, an absolute involved-sHLC value ≥5 g/L was associated with a worse prognosis (PFS, NR vs. 28 months, *P* = 0.016) (Fig 4D). We also observed that patients with involved-sHLC values ≥5 g/L after treatment had a higher level of bone marrow infiltration, as shown with cytology (9.18% vs. 2.27%; *t*-test, *P* = 0.023) and immunophenotyping (1.7% vs. 0.30%; *t*-test, *P* = 0.027).

The suppression of uninvolved-HLC pairs (defined as an abundance less than 50% of the lower concentration in the HLC pair) after treatment did not appear to have a prognostic impact in our series (OS, *P* = 0.466; PFS, *P* = 0.381).

## Discussion

Traditionally, the monoclonal component in serum is quantified and characterized with sPE and immunofixation, respectively. The main limitations of these techniques are the lack of standardization, limited sensitivity, and to a certain extent, subjectivity. sFLC and sHLC assays complement and can increase the diagnostic sensitivity of serum studies in MM.

In addition to the diagnostic utility of the quantitative sFLC assay [3], an abnormal sFLCr or a high content of the involved-sFLC at the treatment response assessment may also be prognostic of MM [3,12]. Moreover, the involved-sFLC concentration also reflects the disease course in the majority of patients. In our series, at the time of diagnosis, HA-sFLCrs (<0.03 or >32) [9] were not associated with differences in prognosis. This finding could be due to the high efficacy of the drugs employed in our clinical trials, which included both bortezomib and immunomodulatory drugs (IMiDe). In this context, classic strategies for evaluating the response have prognostic relevance, and achieving a CR is the main objective for improving survival. In the present study, among patients that achieved CR, the median PFS was 60.7 months and the median OS was NR (*P*<0.0001).

In the entire series of patients, regardless of the response achieved, sFLCr normalization was correlated with a better response (Χ^2^, *P*<0.0001). As expected, a normal sFLCr was correlated with better survival (OS, *P* = 0.025; PFS, *P* = 0.016). However, as we reported previously [15], sFLC normalization in patients that achieved CR did not necessarily indicate a higher quality response, at least from the point of view of clinical significance. Remarkably, we observed that the presence of a very abnormal absolute involved-sFLC value ≥100 mg/L after treatment (median OS, NR vs. 58 months, *P* = 0.03; median PFS, 48 vs. 33 months, *P =* 0.007) occurred more commonly in patients with a partial response. The prognosis of patients in partial response with high levels of involved-sFLC was similar to that of patients with stable disease; thus, these patients might benefit from additional treatment. These findings indicated that the sFLC could be a useful tool for monitoring disease in this scenario.

The greatest value of the recently developed sHLC assays is their ability to differentiate between complex immunoglobulin isotypes; i.e., the heavy chain bound to either a κ or λ light chain (Ig*κ and Ig*λ). This assay facilitates the determination of Ig*-κ/λ ratios (e.g., the IgG-κ/IgG-λ ratio). The strong correlations we found between involved-sHLC values and the MP determined with sPE were highly relevant, both at diagnosis and at the treatment response assessment (*P*<0.01; Pearson’s r = 0.692). This study did not investigate the correlation between sHLC quantification and nephelometry, due to limitations in the nephelometry measurements. When identifying the MP, we could only a quantify the total immunoglobulin content with nephelometry.

In the present study, we observed that HA-sHLCr values (<0.29 or >73) at the time of diagnosis were associated with a shorter PFS, even in the multivariable analysis (S2 Table). Of note, MP quantifications with sPE could not confirm an association between MP values and prognosis in a multivariable analysis, consistent with a previous study [23]. However, in our series, the absolute values of involved-sHLC (for IgG and IgA) showed a linear correlation with absolute values of MP, measured with sPE. This result suggested that sHLC values may represent the tumor burden better than other tests for measuring MPs. Indeed, these measurements could be highly applicable in certain cases, according to a recent report by Michallet et al [24].

When we investigated IgG and IgA isotypes separately, we found a higher correlation between immunoglobulin levels and prognosis in the IgG group than in the IgA group. The discrepancies that we observed between immunoglobulins quantified with sHLC and sPE could be due to co-migration with other proteins in the β region. This factor could have led to inaccurate results, due to the tendency to overestimate with sPE and the possibility of subjectivity in certain cases.

As described above, in our series, at the time of diagnosis, polyclonal immunosuppression (in the form of systemic immunoparesis, immunoparesis of the same class, or both) had no impact on survival, even when we studied values below 50% of the lowest range. Other studies have investigated the role of sHLC assays in MGUS. For example, Katzmann et al. [6] used sHLC assays to measure monoclonal and polyclonal sHLCs separately as a novel means of investigating heavy chain isotype-specific immunoparesis as a predictor of MGUS progression. They found that IgG plasma cell clones suppressed polyclonal IgG synthesis more efficiently than IgA and IgM secretion. More importantly, suppression of an HLC pair (i.e., suppression of IgG-κ in IgG-λ MGUS) was an independent predictor of progression to MM. In our study of patients with symptomatic MM, we found no association between the risk of progression and polyclonal immunosuppression.

When we used sHLC to evaluate the treatment response, measured in terms of sHLCr normalization, we found no differences in survival between responders and non-responders. However, the availability of samples for these measurements was insufficient to allow robust conclusions. In our series, an absolute involved-sHLC value ≥5 g/L implied worse survival, and it was related to a lower level of response. Additionally, among patients with a partial response, an absolute involved-sHLC value ≥5 g/L could identify patients at higher risk of progression. These results suggested that sHLC might be useful for monitoring disease, particularly among patients that are difficult to follow with classical techniques (e.g., when IgA or IgG MM migrates in the β region of the proteinogram). Moreover, we found that absolute involved-sHLC values ≥5 g/L were related to higher levels of plasma cell infiltration, based on cytology (9.18% vs. 2.27%; *t-*test, *P* = 0.023) and immunophenotyping (1.7% vs. 0.30%; *t*-test, *P =* 0.027).

In conclusion, our results suggested that HA-sHLCr values (<0.29 or >73) detected at the time of diagnosis might indicate an increased risk of progression; moreover, HA-sHLCr values might offer better prognostic value than sFLC assays in evaluating patients with MM. After treatment, absolute involved-sHLC values ≥5 g/L could identify patients with poor survival. In our series, the classic CR definition, based on negative immunofixation and bone marrow infiltration <5%, remained a good parameter for assessing treatment response. However, our results showed that, among patients with a poor treatment response, evaluating the response in terms of involved-sFLC and involved-sHLC levels could identify patients with MM that have a poor prognosis. Therefore, these assays are complementary to other techniques for evaluating MP, such as sPE or immunofixation, in cases that are challenging to follow-up (e.g., when IgA MM and IgG MM co-migrate with other proteins in the β region of the proteinogram or in cases of oligosecretory MM). One important consideration is that all of these studies were performed with participants of clinical trials that did not have renal impairment. In particular, renal impairment could interfere with sFLC measurements; therefore, sFLC measurements should be carefully evaluated in real-world practice.

## Supporting information

S1 Table Demographics and baseline characteristics of patients (N = 819). *3 cases were classified as oligosecretory myeloma.
(DOC)

S2 Table Multivariable analysis of prognostic factors measured at diagnosis* (PFS). OR, odds ratio; CI: confidence interval; LDH: lactate dehydrogenase; HA-sHLCr: highly abnormal serum heavy-light chain ratios; FISH: fluorescence in situ hybridization
* Variables initially included: Age, LDH, HA-sHLCr, High vs. low-risk FISH.
(DOC)

S3 Table Relationships between treatment response categories and involved-sHLC levels (<5 or *≥*5 g/L) and sHLC ratios (normal or abnormal). CR: complete response; VGPR: very good partial response; PR: partial response; SD: stable disease; PD: progressive disease
*An involved-sHLC <5 g/L was related to a better treatment response (Χ^*2*^, *P<0*.*0001)*.
**Normalization of the sHLCr was correlated to a better treatment response (Χ^*2*^, *P = 0*.*006)*.
(DOC)

S1 Fig Overall survival (OS) relationships to serum free light chain (sFLC) evaluations. (Figure A in S1 Fig) OS among patients with “highly abnormal” sFLC ratios (<0.03 or >32, red lines, median OS: 71.8 mo) compared to those with normal sFLC ratios (0.03–32, blue lines, median OS: 74.9 mo). (Figure B in S1 Fig) OS among patients with normal (blue, median OS NR) or abnormal (red, median OS: 80 mo) sFLC ratios after treatment, regardless of the response achieved. (Figure C in S1 Fig) OS among patients that achieved complete response (CR) with normal (blue, median OS NR) or abnormal (red, median OS NR) sFLC ratios. (Figure D in S1 Fig) OS among patients with absolute involved-sFLC levels <100 mg/L (blue, median OS NR) or ≥100 mg/L (red, median OS: 64.8 mo).
(TIF)

S2 Fig Overall survival (PFS) relationships to the absolute value of involved serum heavy-light chain (isHLC) and the ratio of highly abnormal sHLC (HA-sHLCr). (Figure A in S2 Fig) OS among patients with absolute values of isHLC <30 g/L (blue, median OS: 67 mo), 30–50 g/L (green, median OS: 57 mo), or >50 g/L (red, median OS: 51 mo). (Figure B in S2 Fig) PFS among patients with HA-sHLCr (<0.29 or >73, red; median OS: 54.4 mo) vs. those with normal sHLCr (0.29–73, blue; median not reached(NR)). (Figure C in S2 Fig) OS among patients with after-treatment absolute values of isHLC <5 g/L (blue, median OS NR) vs. *≥*5 g/L (red, median OS: 57 mo), regardless of the response achieved. (Figure D in S2 Fig) OS among patients that achieved partial response (PR) after treatment with absolute values of isHLC <5 g/L vs. *≥*5 g/L red.
(TIF)
