# Supplementary material for: Prognostic utility of serum free light chain ratios and heavy-light chain ratios in multiple myeloma in three PETHEMA/GEM phase III clinical trials
Source: PLoS One. 2018 Sep 7;13(9):e0203392. doi: 10.1371/journal.pone.0203392 (PMC6128544; doi:10.1371/journal.pone.0203392)
Supplement: S1 Table — *3 cases were classified as oligosecretory myeloma. (DOC) [file pone.0203392.s001.doc]

| **Characteristics** |  | **GEM05<65 y** | **GEM05>65 y** | **GEM2010>65 y** | **All** |
| --- | --- | --- | --- | --- | --- |
| No. |  | n=331 | n=254 | n=234 | N=819 |
| Age (years) |  |  |  |  |  |
| median |  | 57 | 73 | 75 | 69 |
| (range) |  | (25–66) | (66–85) | (65–88) | (25–89) |
| Sex, male/female (%) |  | 170/161 | 122/132 | 125/109 | 417 (50.9%)/ 402 (49.1%) |
| β2 microglobulin (mg/dL) | |  |  |  |  |
| median |  | 3.1 | 3.9 | 4.2 | 3.6 |
| (range) |  | (0.18–21.50) | (0.20–21.70) | (0.15–22.10) | (0.15–22.10) |
| Albumin (mg/dL) |  |  |  |  |  |
| median value |  | 3.69 | 3.5 | 3.5 | 3.6 |
| (range) |  | (1.70–5.50) | (1.43–5.24) | (1.8–5.10) | (1.43–5.50) |
| FLC ratio at diagnosis, n |  | n=238 | n=224 | n=161 | N=623 |
| median |  | 4.63 | 4.18 | 4 | 4.3 |
| (range) |  | (0–48000) | (0–25662.1) | (0–30300) | (0–48000) |
| HLC ratio at diagnosis, n |  | n=45 | n=42 | n=96 | N=183 |
| median |  | 16.6 | 24.8 | 10.395 | 16.6 |
| (range) |  | (0.01–525) | (0.002–706.024) | (0–3976.88) | (0–3976.87) |
| Myeloma subtype, n (%) |  | n=331 (%) | n=254 (%) | n=231 (%) * | N=816 (%) * |
| IgG κ |  | 131 (39.6) | 97 (38.2) | 74 (32) | 302 (37) |
| IgG λ |  | 73 (22.1) | 53 (20.9) | 50 (21.6) | 176 (21.6) |
| IgA κ |  | 42 (12.7) | 44 (17.3) | 45 (19.5) | 131 (16.1) |
| IgA λ |  | 33 (10) | 32 (12.6) | 33 (14.1) | 98 (12) |
| IgM κ |  | 1 (0.3) | – | – | 1 (0.1) |
| IgM λ |  | 6 (1.8) | – | 1 (0.4) | 7 (0.9) |
| Light chain κ |  | 25 (7.6) | 12 (4.7) | 15 (6.5) | 52 (6.3) |
| Light chain λ |  | 19 (0.3) | 16 (6.3) | 13 (5.6) | 48 (5.9) |
| ISS stage, n (%) |  |  |  |  |  |
| I |  | 130 | 64 | 52 | 246 (30.3%) |
| II |  | 134 | 104 | 102 | 340 (41.9%) |
| III |  | 67 | 85 | 73 | 225 (27.7%) |
| Median follow-up, months (range) | | 56.6  (1.27–96.4) | 48.7  (0.23–947.) | 30.2  (0.43–69.7) | 45.96  (0.23–96.4) |
| Cytogenetics, n (%) |  |  |  |  |  |
| Standard risk/High risk, based on [t(4;14). del(17p)] | | 183/53 | 192/47 | 121/37 | 496 (78.4%)/137 (21.6%) |
