# Supplementary material for: Prognostic utility of serum free light chain ratios and heavy-light chain ratios in multiple myeloma in three PETHEMA/GEM phase III clinical trials
Source: PLoS One. 2018 Sep 7;13(9):e0203392. doi: 10.1371/journal.pone.0203392 (PMC6128544; doi:10.1371/journal.pone.0203392)
Supplement: S2 Table — OR, odds ratio; CI: confidence interval; LDH: lactate dehydrogenase; HA-sHLCr: highly abnormal serum heavy-light chain ratios; FISH: fluorescence in situ hybridization * Variables initially included: Age, LDH, HA-sHLCr, High vs. low-risk FISH. (DOC) [file pone.0203392.s002.doc]

| **Variable** | ***P*; OR [95% CI]** |
| --- | --- |
| **Age** | 0.003; 1.04 [1.01–1.06] |
| **LDH** | 0.03; 0.4 [0.26–0.94] |
| **HA-sHLCr** | 0.01; 1.78 [1.14–2.78] |
| **High- vs. low-risk FISH** | 0.02; 1.75 [1.11–2.74] |
