# Supplementary material for: Prognostic utility of serum free light chain ratios and heavy-light chain ratios in multiple myeloma in three PETHEMA/GEM phase III clinical trials
Source: PLoS One. 2018 Sep 7;13(9):e0203392. doi: 10.1371/journal.pone.0203392 (PMC6128544; doi:10.1371/journal.pone.0203392)
Supplement: S3 Table — CR: complete response; VGPR: very good partial response; PR: partial response; SD: stable disease; PD: progressive disease *An involved-sHLC <5 g/L was related to a better treatment response (Χ2, P<0.0001). **Normalization of the sHLCr was correlated to a better treatment response (Χ2, P = 0.006). (DOC) [file pone.0203392.s003.doc]

|  | **Involved-serum HLC (%)** | | **Serum HLC ratio (%)** | |  |
| --- | --- | --- | --- | --- | --- |
| **Response** | *<5 g/L** | *≥5 g/L* | *Normal*** | *Abnormal* | *Total (100%)* |
| **CR** | *23 (92)* | *2 (8)* | *13 (52)* | *12 (48)* | *25* |
| **VGPR** | *21 (88)* | *3 (12)* | *8 (33)* | *16 (66)* | *24* |
| **PR** | *13 (56)* | *20 (44)* | *4 (12)* | *29 (88)* | *33* |
| **SD** | *0* | *5 (100)* | *0* | *5 (100)* | *5* |
| **PD** | *0* | *2(100)* | *0* | *2 (100)* | *2* |
| **Total** | *57* | *32* | *25* | *64* | *89* |
