# Supplementary figures and images for: Prognostic utility of serum free light chain ratios and heavy-light chain ratios in multiple myeloma in three PETHEMA/GEM phase III clinical trials
Source: PLoS One. 2018 Sep 7;13(9):e0203392. doi: 10.1371/journal.pone.0203392 (PMC6128544; doi:10.1371/journal.pone.0203392)

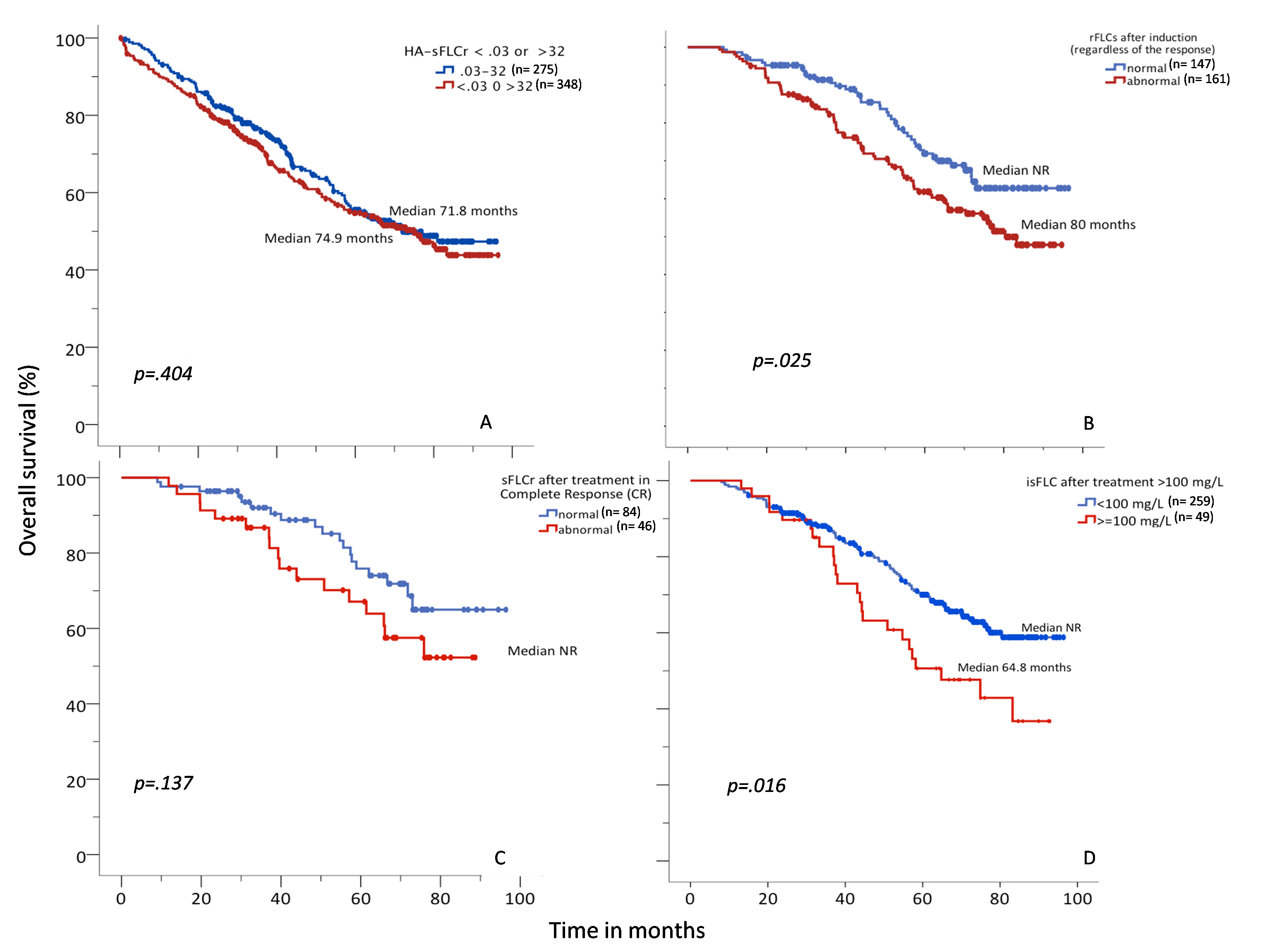

Supplement: S1 Fig — (Figure A in S1 Fig) OS among patients with “highly abnormal” sFLC ratios (<0.03 or >32, red lines, median OS: 71.8 mo) compared to those with normal sFLC ratios (0.03–32, blue lines, median OS: 74.9 mo). (Figure B in S1 Fig) OS among patients with normal (blue, median OS NR) or abnormal (red, median OS: 80 mo) sFLC ratios after treatment, regardless of the response achieved. (Figure C in S1 Fig) OS among patients that achieved complete response (CR) with normal (blue, median OS NR) or abnormal (red, median OS NR) sFLC ratios. (Figure D in S1 Fig) OS among patients with absolute involved-sFLC levels <100 mg/L (blue, median OS NR) or ≥100 mg/L (red, median OS: 64.8 mo). (TIF) [file pone.0203392.s004.TIF]

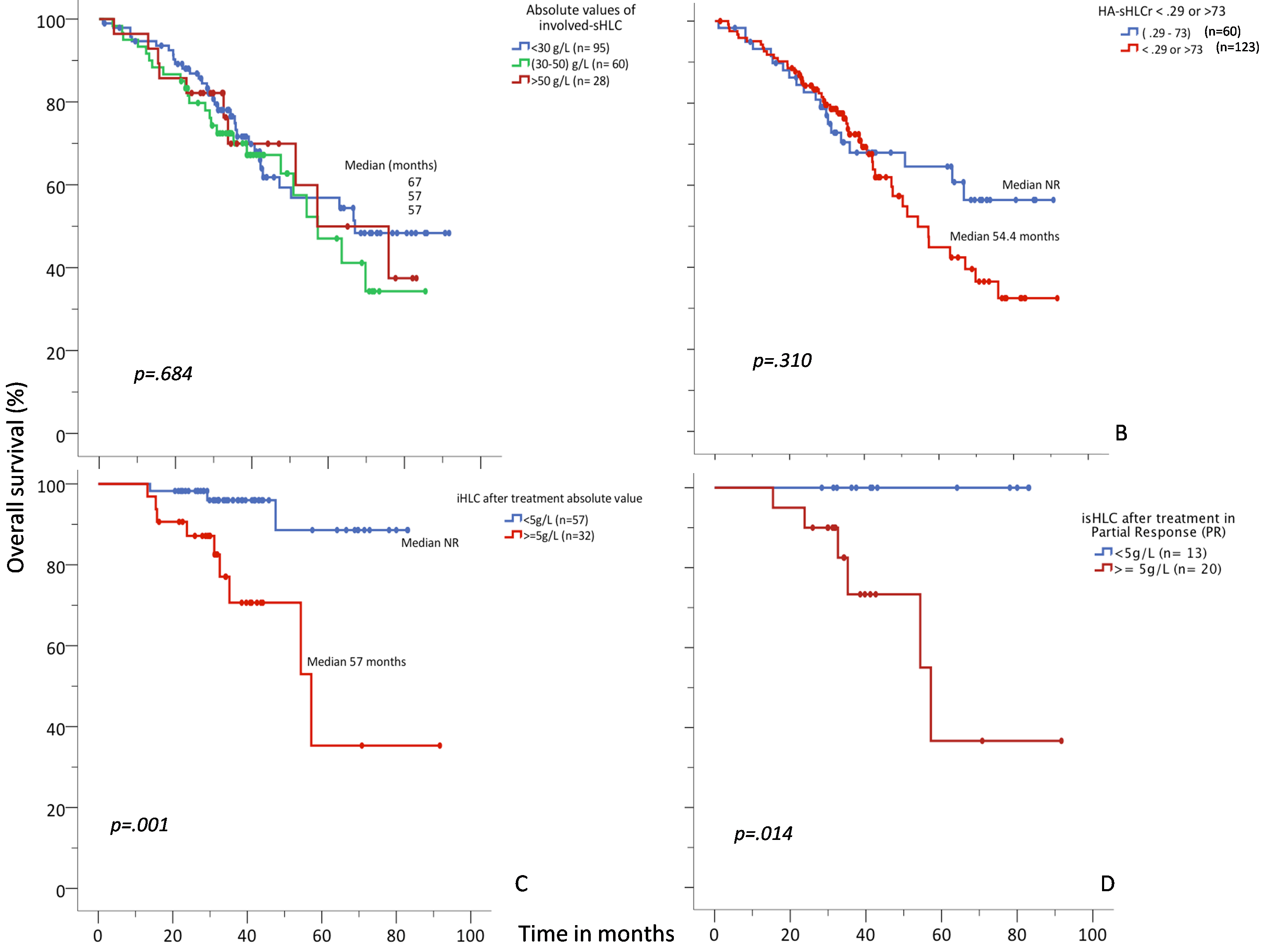

Supplement: S2 Fig — (Figure A in S2 Fig) OS among patients with absolute values of isHLC <30 g/L (blue, median OS: 67 mo), 30–50 g/L (green, median OS: 57 mo), or >50 g/L (red, median OS: 51 mo). (Figure B in S2 Fig) PFS among patients with HA-sHLCr (<0.29 or >73, red; median OS: 54.4 mo) vs. those with normal sHLCr (0.29–73, blue; median not reached(NR)). (Figure C in S2 Fig) OS among patients with after-treatment absolute values of isHLC <5 g/L (blue, median OS NR) vs. ≥5 g/L (red, median OS: 57 mo), regardless of the response achieved. (Figure D in S2 Fig) OS among patients that achieved partial response (PR) after treatment with absolute values of isHLC <5 g/L vs. ≥5 g/L red. (TIF) [file pone.0203392.s005.TIF]
